# Inference and prioritization of tissue-specific regulons in *Arabidopsis* and *Oryza*

**DOI:** 10.1007/s42994-024-00176-2

**Published:** 2024-07-16

**Authors:** Honggang Dai, Yaxin Fan, Yichao Mei, Ling-Ling Chen, Junxiang Gao

**Affiliations:** 1 Hubei Key Laboratory of Agricultural Bioinformatics, College of Informatics, Huazhong Agricultural University, Wuhan, 430070 China; 2 State Key Laboratory for Conservation and Utilization of Subtropical Agro-bioresources, College of Life Science and Technology, Guangxi University, Nanning, 530004 China

**Keywords:** Transcriptional regulation, Graph convolutional network, Tissue-specific, Regulon, Transcription factor

## Abstract

A regulon refers to a group of genes regulated by a transcription factor binding to regulatory motifs to achieve specific biological functions. To infer tissue-specific gene regulons in *Arabidopsis*, we developed a novel pipeline named InferReg. InferReg utilizes a gene expression matrix that includes 3400 *Arabidopsis* transcriptomes to make initial predictions about the regulatory relationships between transcription factors (TFs) and target genes (TGs) using co-expression patterns. It further improves these anticipated interactions by integrating TF binding site enrichment analysis to eliminate false positives that are only supported by expression data. InferReg further trained a graph convolutional network with 133 transcription factors, supported by ChIP-seq, as positive samples, to learn the regulatory logic between TFs and TGs to improve the accuracy of the regulatory network. To evaluate the functionality of InferReg, we utilized it to discover tissue-specific regulons in 5 *Arabidopsis* tissues: flower, leaf, root, seed, and seedling. We ranked the activities of regulons for each tissue based on reliability using Borda ranking and compared them with existing databases. The results demonstrated that InferReg not only identified known tissue-specific regulons but also discovered new ones. By applying InferReg to rice expression data, we were able to identify rice tissue-specific regulons, showing that our approach can be applied more broadly. We used InferReg to successfully identify important regulons in various tissues of *Arabidopsis* and *Oryza*, which has improved our understanding of tissue-specific regulations and the roles of regulons in tissue differentiation and development.

**Supplementary Information:**

The online version contains supplementary material available at 10.1007/s42994-024-00176-2.

## Introduction

Transcription factors (TFs) are proteins that bind to specific DNA regions and can activate or inhibit other genes, known as target genes (TGs). When a transcription factor interacts with its target genes, it forms a regulon, which is responsible for carrying out specific biological functions by modifying the expression of the target genes (Trefflich et al. 2020). Researchers have identified regulons that are uniquely expressed in specific plant tissues, playing a critical role in orchestrating the development of corresponding organs. Identifying tissue-specific regulons and genes related to important traits is a crucial task in molecular design breeding (Jing et al. 2021). Gene transcription and translation require materials and energy. Studies focus on tissue-specific expression and regulation of agronomic traits to improve material and energy utilization efficiency. Researchers combine tissue-specific promoters with the expression regions of target genes to construct transgenic biological models. This allows certain genes to be expressed only in specific tissues or organs while displaying traits like high yield, insect resistance, and disease resistance. Alanine plays a crucial role in plant nitrogen metabolism. The enzyme AlaAT (alanine aminotransferase) is involved in the synthesis and degradation of alanine through the reverse reaction. Researchers overexpressed AlaAT in transgenic rice using the root-specific promoter (OsAnt1) to develop varieties with efficient nitrogen utilization. As a result, transgenic plants produced more tillers and exhibited more vigorous growth than the control plants when grown in a hydroponic solution containing ammonium as a nitrogen source. This improvement led to a significant increase in the biomass and yield of rice (Shrawat et al. 2008). In a previous study, a Cre/loxP-based strategy was developed to control the expression of transgenes in green tissues while preventing their expression in nongreen tissues. The study found that the expression level and concentration of Cry1Ab/c was high in green tissues such as leaves and stems, but low or undetectable in root and kernel tissues. The transgenic plants showed high resistance to two common lepidopteran pests, *Ostrinia furnacalis* and *Spodoptera frugiperda*, in comparison to the control group (Yuan et al. 2023). Recent studies have also used CRISPR/Cas9 technology to accurately modify the promoter regions or regulatory elements of target genes in crops. This modification can either enhance or reduce the activity of promoters, allowing for the regulation of gene expression in specific tissues (Kong et al. 2023). Systematic exploration of tissue-specific regulons offers the potential in developing regulatory systems to control TGs or aid in the production of necessary metabolic products in specific organs and tissues.

Efforts over the past two decades have generated thousands of transcriptome data from different tissues of *Arabidopsis*, and the situation is similar for other model plants. The full utilization of this data can enhance the understanding of tissue-specific regulons. GENIE3 (Huynh-Thu et al. 2010) and SCENIC (Van de Sande et al. 2020) are frequently used to infer regulatory relationships from expression profiles. GENIE3 uses co-expression information from transcriptome data to identify regulatory relationships between genes. However, this approach can introduce false positives. Previous studies have shown that gene regulation is the result of the joint action of multiple omics. Relying solely on a single type of data makes it challenging to characterize complex life processes. SCENIC incorporates DNA motif information into co-expression to infer gene regulatory relationships. It then examines the activity of transcription factor gene sets to identify cell states. SCENIC has been utilized in numerous studies. However, it is specifically tailored for single-cell transcriptome analysis and is directly applicable only to humans, mice, and Drosophilas.

Single-cell omics can highlight distinctions between individual cells, aiding researchers in comprehending cellular heterogeneity, encompassing variations in development, disease, and other biological processes (Ferrari et al. 2022). However, the development of single-cell omics in plants significantly lags behind that in humans and animals. Using single-cell omics data to identify regulatory relationships still faces many challenges, including processing plant cell walls, inferring marker genes, identifying cell types, and standardizing data analysis. The specific details of single-cell omics in plants are further discussed in the discussion section. Since there is still limited plant single-cell omics data, this study utilized 3400 bulk expression profiles and Chip-seq data to analyze *Arabidopsis* tissue-specific regulons. Another area for improvement is effectively extracting and utilizing network structure and node interaction information. Graph convolutional network (GCN) offers significant advantages in graph representation, node classification, node prediction, link classification, and link prediction (Wu et al. 2021). The main idea behind GCNs is to create representations for nodes and edges, and then learn the connections between nodes through message-passing mechanisms. Notably, GCN makes full use of the inherent structural information in graph data (Nikolentzos et al. 2020), which makes it well-suited for inferring regulatory relationships. The graph convolutional network updates the node representation by gathering information from neighboring nodes. This enables the gene representation to capture local information within the graph structure. It learns node representations by propagating information on the graph structure. Each gene can exchange and aggregate information with its neighboring nodes during information propagation, gradually updating its representation. Therefore, graph convolutional network considers both the local neighbor information of nodes and the global structural information of the entire graph. A study used GCN on *Arabidopsis* data and successfully predicted gene expression within and across species (Ferebee and Buckler 2023). Furthermore, GCNs have been widely used in various bioinformatics tasks, such as predicting protein–protein interaction and discovering new drugs (Jha et al. 2022; Li et al. 2022b).

In our research, we introduced a new method called InferReg, which aims to identify specific regulatory networks in *Arabidopsis* tissues. We used a broad set of 3400 transcriptomic datasets to create networks based on gene expression. To enhance the reliability of these networks, we included TFBS enrichment analysis, which helps eliminate incorrect interactions based only on expression data. Additionally, we gathered ChIP-seq data for 133 TFs to support our findings. We used the interactions between transcription factors and target genes as input to create a GCN for understanding the regulatory relationships between TFs and TGs. We identified groups of TFs and their TGs, called regulons, which have important functions in specific tissues. Then, we assigned regulons to different types of tissue, ranked them, and compared the top-ranked regulons with the *Arabidopsis* Genome-Wide Association Studies database (AraGWAS) and existing literature (Togninalli et al. 2017). The results show that our pipeline can effectively identify many tissue-specific regulons with significant biological functions. To test if InferReg is applicable to other cases, we used it on rice even though there is limited ChIP-seq data available. Surprisingly, InferReg was able to identify tissue-specific regulons in rice that have meaningful biological implications.

## Results

### Overview of InferReg

**Fig. 1:**
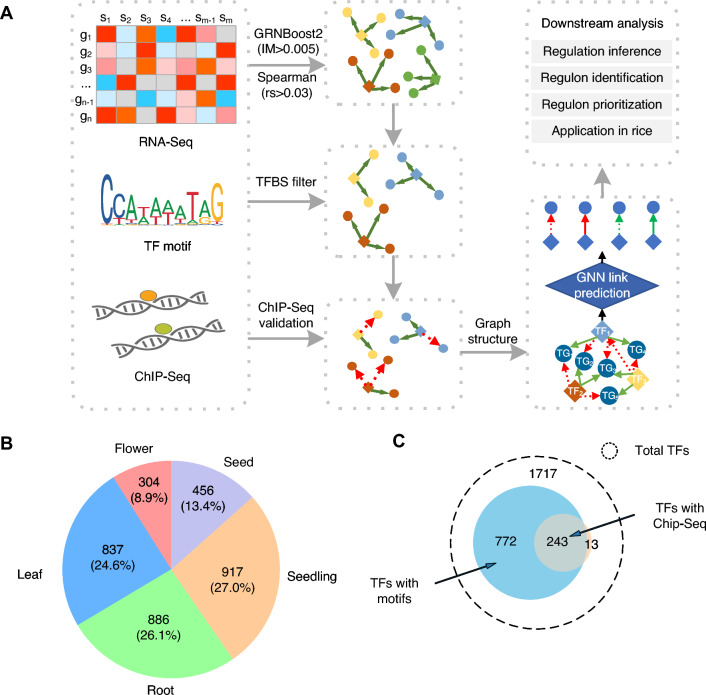
Overview of the InferReg workflow and dataset summary of *Arabidopsis*. **A** InferReg workflow diagram. The left panel displays the input data, including RNA-seq, TFBS, and TF binding profiles obtained from ChIP-seq. The middle panel outlines three steps: co-expression genes extraction, TFBS enrichment, and ChIP-seq validation. The right panel represents downstream analysis using GCN. **B** The number of *Arabidopsis* RNA-seq samples was distributed across five tissues: root, leaf, flower, seed, and seedling in InferReg. **C** Number of TFs of *Arabidopsis* used in InferReg. The dashed circle represents the total number of TFs

GCNs can aggregate local information into global information, effectively handling complex regulatory relationships and exhibiting strong representation and prediction capabilities. Therefore, we developed InferReg as an integrated GCN that combines information derived from gene expression and TF binding motif data to infer tissue-specific regulons (Fig. 1A). Initially, InferReg used GRNBoost2 (Moerman et al. 2019) and the expression matrix to calculate a preliminary co-expression network. This network provides potential regulatory relationships between genes. Subsequently, these initial regulatory relationships undergo further filtering and validation using Spearman correlation coefficients, TF binding site, and ChIP-seq data.

### Extraction of regulatory relationships from multi-omics data

We have selected 3400 high-quality datasets out of 20,068 datasets available in the database. These datasets cover five different tissues: 304 for flower, 837 for leaf, 886 for root, 917 for seed, and 456 for seedling (Supplementary Table S1). Each sample provided expression values for 37,886 genes, resulting in a 37,886 × 3400 input expression matrix for InferReg (Fig. 1B). We have identified 1868 unique binding sites represented by position weight matrices (PWMs) and mapped them to 1015 *Arabidopsis* TFs (Fig. 1C).

InferReg first analyzed the initial regulatory relationships using importance scores (IM) from GRNBoost2. We set a threshold of 0.005 to filter out weak regulatory relationships and kept only the stronger associations, resulting in 929 TFs with an average of 7196 putative TGs per TF. We then calculated the Spearman correlation coefficient across different samples for each initial regulatory relationship, focusing on those with a correlation coefficient greater than 0.03. This narrowed down the number of potential TGs decreased from 7196 to 4928, resulting in an average of 4,928 potential TGs per TF. The affinity of TFs for TGs plays a crucial role in regulating gene expression (Fletcher 2023). We conducted a search for potential binding sites near the transcription start site (TSS) of TGs using the available TFBS data. To overcome the issue of incomplete TFBS information for known TFs, we employed a strategy of sharing TFBS among TFs belonging to the same family, thus expanding the available information (Suvorova et al. 2015). As a result, the number of TFs decreased from 929 to 720, and the average number of potential TGs per TF was also reduced to 3858.

**Fig. 2:**
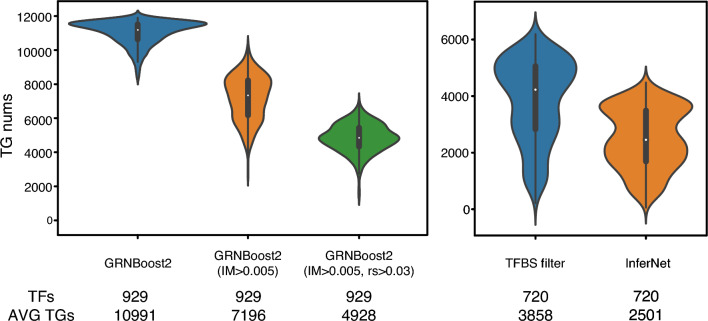
Distribution of TFs involved in gene regulation and their TG numbers after five steps: GRNBoost2 computation, IM (Importance) filtering, Spearman correlation coefficient filtering, TFBS filtering, and GCN inference

To confirm the regulatory relationships between TFs and TGs, we used ChIP-seq data for 256 *Arabidopsis* TFs to observe the interaction between DNA and proteins (Wilson et al. 2010) (Supplementary Table S2). By integrating RNA-seq expression data with TFBS enrichment and ChIP-seq validation, we identified 133 TFs and their TGs, with an average of 365 TGs per TF. These regulatory relationships can be considered as positive (Fig. 2).

### Construction and performance of GCN

The regulatory relationships deduced solely from these experimental data may not fully capture the intricate interactions among genes. To overcome this limitation, we have introduced a supervised GCN. GCN has the capability to utilize the comprehensive global structural information of the network by aggregating neighborhood details, thus enabling the capture of indirect interactions and complex regulatory patterns among genes. We utilized the filtered regulatory relationships as input, with gene expression levels as node features, and the known regulatory relationships as the ground truth. The model training was carried out on a system with an NVIDIA GeForce RTX 3080 GPU (10 GB VRAM), an Intel(R) Xeon(R) Silver 4208 CPU (2.10 GHz), and 32 GB of RAM. InferReg was able to uncover more complex regulatory patterns among TFs and TGs and measure the strength of these regulatory connections, ultimately enhancing the accuracy of ranking regulatory relationships.

**Fig. 3:**
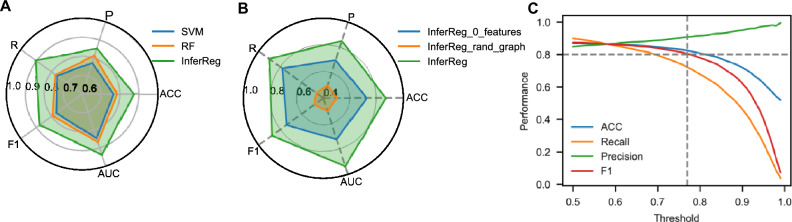
Model performance evaluation. **A** Performance comparison of InferReg with SVM and RF models on five metrics: accuracy, precision, recall, F1, and AUC. **B** Performance of InferReg, InferReg_0_features (InferReg with zeroed node features), and InferReg_rand_graph (InferReg with randomized graph). **C** Performance trends of InferReg in accuracy, recall, precision, and F1 at different classification thresholds

We assessed the performance using accuracy, precision, recall, F1 score, and AUC metrics. The AUC achieved a value of 0.94, whereas the F1 score reached 0.88, demonstrating excellent classification performance. To further validate its superiority, we compared InferReg with commonly used benchmark models in supervised learning, namely Support Vector Machine (SVM) (Vapnik 1995) and Random Forest (RF) (Breiman 2001). The results show that InferReg outperformed both SVM and RF in terms of classification (Fig. 3A). SCENIC is specifically designed for single-cell data and cannot be used for bulk expression profiles (Van de Sande et al. 2020). GENIE3 infers regulatory relationships solely from transcriptome data (Huynh-Thu et al. 2010), while DeepTFni relies only on ATAC-seq data for inference (Li et al. 2022a). On the other hand, CellOracle (Kamimoto et al. 2023) is similar to our study as it can infer regulatory relationships from RNA-seq data and transcription factor binding sites. Therefore, we compared InferReg with CellOracle using the same gold standard dataset. The results showed an F1 score of 0.81 for InferReg and 0.26 for CellOracle. It is important to note that InferReg is supervised whereas CellOracle is unsupervised, making a fair comparison of their performance challenging. Nevertheless, the outcomes still demonstrate the advantage of InferReg in inferring regulatory relationships.

The graph neural network includes both node feature information and graph structure information. The node feature information describes the attributes of each node as a vector consisting of the expression level of genes in each sample. The graph structure information defines the relationships between nodes. We carried out two additional experiments to assess the significance of both the graph structure and node features in predicting results. In the first, we shuffled the links in the graph randomly to mask the graph structure, while retaining only the node feature information. The results showed a significant impact on performance, with an AUC of 0.51. This was due to the fact that random link changes disrupted the propagation of structure information. In the second experiment, we retained the original graph structure but set all node features to zero. This was done to mask node feature information and observe whether the model could learn relationships solely from graph structure. We noticed a notable drop in performance (AUC = 0.72) (Fig. 3B). The results of the experiments suggest that both node feature and graph structure have an impact on model performance. Additionally, we implemented a post-processing strategy, which improved the accurate identification of regulatory relationships (Fig. 3C).

### Identification of tissue-specific regulons

**Fig. 4:**
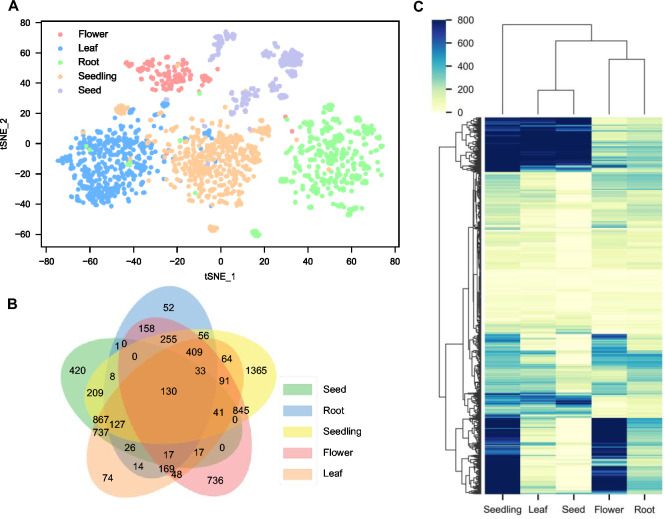
Tissue specificity in *Arabidopsis*. **A** t-SNE visualization of expression patterns of RNA-seq samples from five tissues. **B** Venn diagram of differentially expressed genes in five tissues. **C** Clustered heatmap of the number of genes in regulatory regions of five tissues

The composition of regulons varies significantly in different tissues. Therefore, we wanted to find out if InferReg can identify which genes, transcription factors, and functional modules are present in a specific tissue and play a crucial role. To examine the differences in gene expression patterns across different tissues, we visually analyzed RNA-seq expression profiles for five tissues using t-SNE (Fig. 4A). The results showed that samples from the same tissue tended to cluster together. In contrast, different tissues could be clearly distinguished. These findings emphasize the existence of significant tissue-specific expression patterns.

We conducted an analysis to identify genes that are differentially expressed in different tissues, to understand the specific regulatory relationships within each tissue. In Fig. 4A, B Venn diagram shows the number of differentially expressed genes (DEGs) in the flower, leaf, root, seed, and seedling tissues. We identified 2916, 2864, 1455, 4500, and 6410 DEGs in these tissues, respectively. Next, we determined tissue-specific regulons by analyzing the predicted regulons from the GCN for each tissue. The regulatory interactions in a regulon can involve multiple genes that may be expressed simultaneously or sequentially under different environmental conditions, enabling complex regulation of cellular functions (Trefflich et al. 2020). We identified DEGs in each tissue compared to other tissues and selected TFs and their corresponding TGs from these genes. These TFs and TGs were combined to form tissue-specific regulons (Supplementary Table S3).

In Fig. 4C, there is a clustering heatmap showing how 720 TFs are distributed in the inferred regulons across five tissues. The colors on the heatmap represent the number of TGs regulated by each TF, indicating the activity level of the regulons. These results demonstrate the participation of TFs and their regulons in tissue-specific regulation, revealing the complex and diverse transcriptional regulation involved in plant development.

### Prioritization of tissue-specific regulons

Table 1 provides detailed statistics on the relevant genes and TFs of five plant tissues. In the RNA-seq expression matrix, the five tissues show 421, 699, 354, 405, and 474 tissue-specific genes, respectively. Among these genes, 108, 153, 104, 72, and 176 TFs are associated with the respective tissues, with most of these TFs being involved in corresponding tissue-specific regulons.

**Table 1:** Number of genes associated with GO enrichment analysis in five *Arabidopsis* tissues

Tissue	Tissue-associated genes (total/expressed)	Tissue-associated TFs	Regulating TF (only as TF/only as TG)
Flower	1095/421	108	74/11
Leaf	1422/699	153	121/19
Root	753/354	104	93/0
Seed	1199/405	72	39/6
Seedling	1118/474	176	128/12

**Fig. 5:**
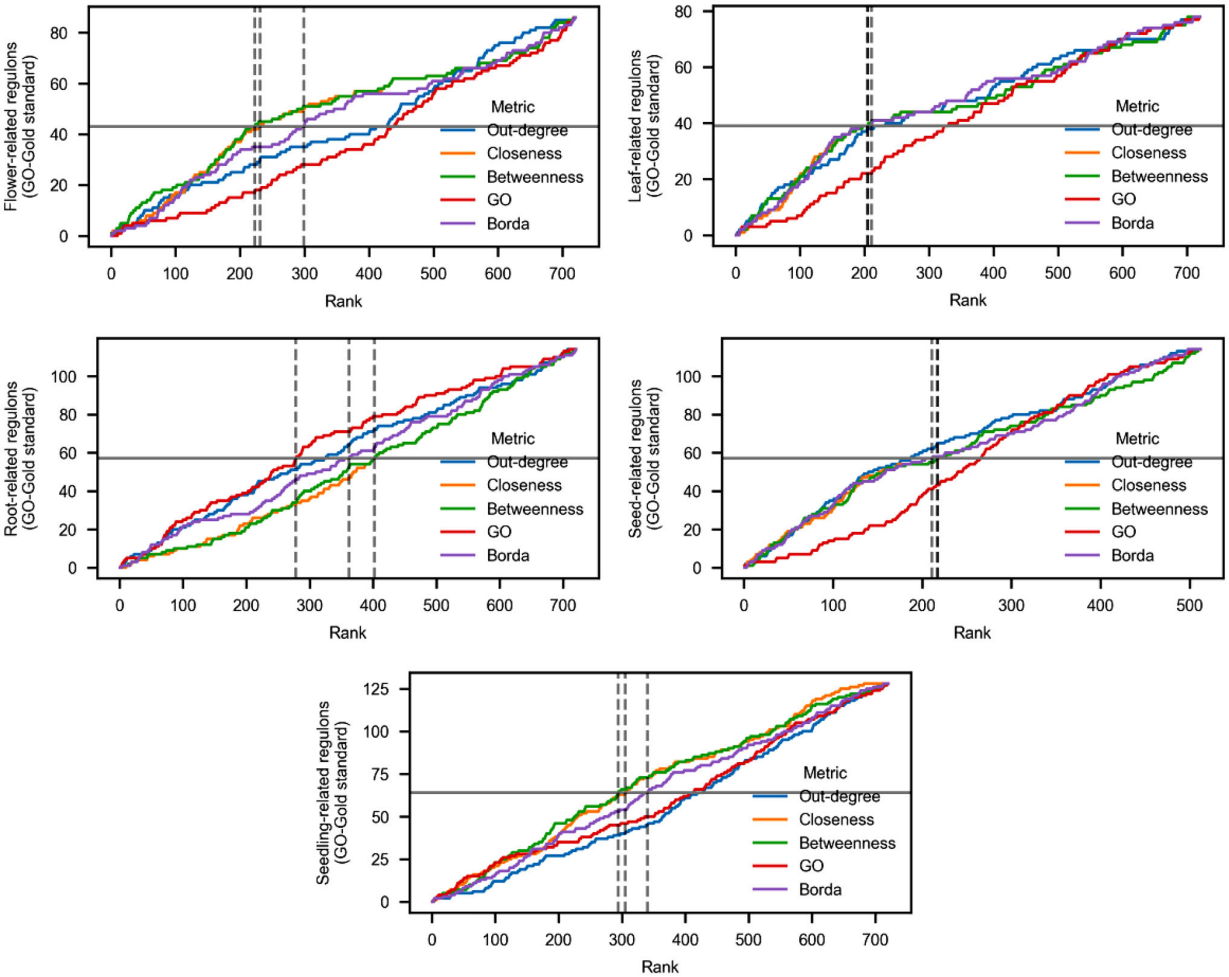
Evaluation of ranking metrics for regulatory regions in five tissues. The curve represents the number of known regulons with GO gold standards among a specific quantity of top-ranked regulons in each tissue, based on different metrics. The x-axis represents the number of top-ranked regulons according to each metric, and the y-axis represents the number of known functional standards among the corresponding ranked regulons. In each plot, the horizontal line represents half of the gold standard regulons for that tissue, and the vertical line represents the ranking (R50) at which half of the gold standard dataset is retrieved for each metric

Given the substantial number of regulons inferred by InferReg, we sought to identify the TFs exerting a more significant influence in tissue-specific regulons. To achieve this, we utilized network centrality measures, encompassing closeness centrality, betweenness centrality, out-degree, and functional specificity, to prioritize these regulons. Initially, the subnetworks were assessed and individually ranked based on these four metrics. Subsequently, we calculated the geometric mean of these rankings using the Borda count method to establish a final prioritization order (Table 2, Fig. 5).

**Table 2:** Mean and standard deviation of rankings for five metrics in five tissues

Tissue	Out_deg	Closeness	Betweenness	GO	Borda
Flower	4	2	1	5	3
Leaf	4	3	2	5	1
Root	3	4	5	1	2
Seed	5	1	3	4	2
Seedling	5	1	2	4	3
Average	4.2	2.2	2.6	3.8	2.2
SD	0.84	1.30	1.52	1.64	0.84

We further analyzed the top-ranked regulons to validate their biological significance. Initially, we gathered genes linked to leaf, root, and seed phenotypes from the AraGWAS database (Togninalli et al. 2017). We identified 66, 241, and 39 genes associated with leaf, root, and seed traits, respectively (Supplementary Table S4). Out of these genes, 25, 97, and 15 were expressed in the RNA-seq dataset. Among the 9 TFs associated with root phenotypes, 3 TFs served as the primary regulators in the inferred regulons. Additionally, we examined the distribution of phenotype-related genes in the inferred root regulons. The findings revealed that 491 (68.38%) of inferred root regulons contained phenotype-related genes. Furthermore, out of the top 100 regulons, 16, 29, 21, 7, and 19 were regulated by TFs from the AraGWAS gold standard in flowers, leaves, roots, seeds, and seedlings, respectively. This finding emphasizes the important biological significance of the inferred tissue-specific regulons, with a significant number being linked to specific plant tissues.

The top-ranked regulons consist of many GO gold standard regulons. We also analyzed regulons that ranked highly but were not part of the GO gold standard and identified several tissue-specific TFs that play crucial biological roles. For example, MYB56 (ranked first) in the root has been validated as an R2R3 MYB family TF that exhibits specific expression in the root. This TF inhibits cell division in the root apical meristem and the quiescent center (QC). MYB56 collaborates with BES1 to suppress cell division in the root apical meristem, thereby maintaining the characteristics of root stem cells (Vilarrasa-Blasi et al. 2014).

In addition, MYB56 controls how roots respond to sodium, lithium ions, and oxidative stress (Matsuo et al. 2015). HFR1, ranked first, shows specific expression in leaves, is a regulator of photomorphogenesis through the brassinosteroid signaling pathway (Fairchild et al. 2000). MYB88, ranked 20th, shows specific expression in long siliques and directly activates downstream genes to regulate the normal dehiscence process of the long siliques. In the constructed network, MYB88 also shows a distinct high-expression pattern in the flower (Lal and Das 2016). The biosynthesis of flavanols in different parts of *Arabidopsis* has been thoroughly studied (Stracke et al. 2007, 2010). Hence, we also investigated whether InferReg identified MYB regulons linked to this biological process. The findings revealed that MYB11 and MYB12 are expressed exclusively in roots, ranking 27th and 4th, respectively. MYB111, another crucial player, was discovered in leaves, ranking 169th out of a total of 720 factors. Additionally, MYB75, a significant TF involved in anthocyanin synthesis predominantly found in leaves, held the 20th position in the ranking. These results show that InferReg is capable of identifying potential tissue-specific regulons that have significant biological functions.

### Application of InferReg in rice

To evaluate the generalization ability of InferReg, we used this pipeline on rice. We collected various multi-omics data of *Nipponbare*, which included 833 RNA-seq datasets from the Plant Public RNA-seq Database. These datasets covered five distinct tissues: 164 from shoot, 409 from leaf, 76 from flower, 138 from root, and 46 from seedling (Yu et al. 2022) (Supplementary Table S5). Additionally, we used 1862 TFs from the PlantTFDB database (Jin et al. 2016) and 1580 TFBSs from the CisBP database (Weirauch et al. 2014). The data were preprocessed and utilized using a similar approach to the previous analysis conducted in *Arabidopsis*. With these data, a GRN comprising 530 TFs was constructed. These TFs accounted for 63% of the total TFs in the *Nipponbare* expression atlas (530 out of 848). On average, each TF had 3991 TGs. In comparison to *Arabidopsis*, the available TFBS data for rice was more limited, with 1580 TFBSs for rice and 1868 TFBSs for *Arabidopsis*, respectively.

Due to the limited availability of ChIP-seq data for rice, we were only able to collect ChIP-seq data for 17 rice TFs (Fu et al. 2022). To address this limitation, we utilized the pre-trained *Arabidopsis* GCN and applied it to rice, then analyzed the resulting predictions. The biological basis of this is as follows: many TFs are relatively stable in the evolutionary process of plants, especially those that play critical roles in fundamental biological processes. This stability is evident in their sequence composition, protein structure, binding sites, functions, and regulatory mechanisms of transcription factors (Feng et al. 2020; Katz et al. 2022). Throughout a long evolutionary process, their actions and regulatory modes have shown similarity. Additionally, certain fundamental signaling pathways and regulatory relationships are relatively conservative. Common hormone signaling pathways, such as gibberellin, abscisic acid, and agonists, exist in *Arabidopsis* and rice. Furthermore, our study, as well as some other studies (Ferebee and Buckler 2023; Mourad 2023), have employed a similar strategy. For instance, Mourad et al. successfully predicted regulatory elements in the mouse genome using GNN models trained on human gene expression data and ChIP-seq datasets (Mourad 2023).

**Fig. 6:**
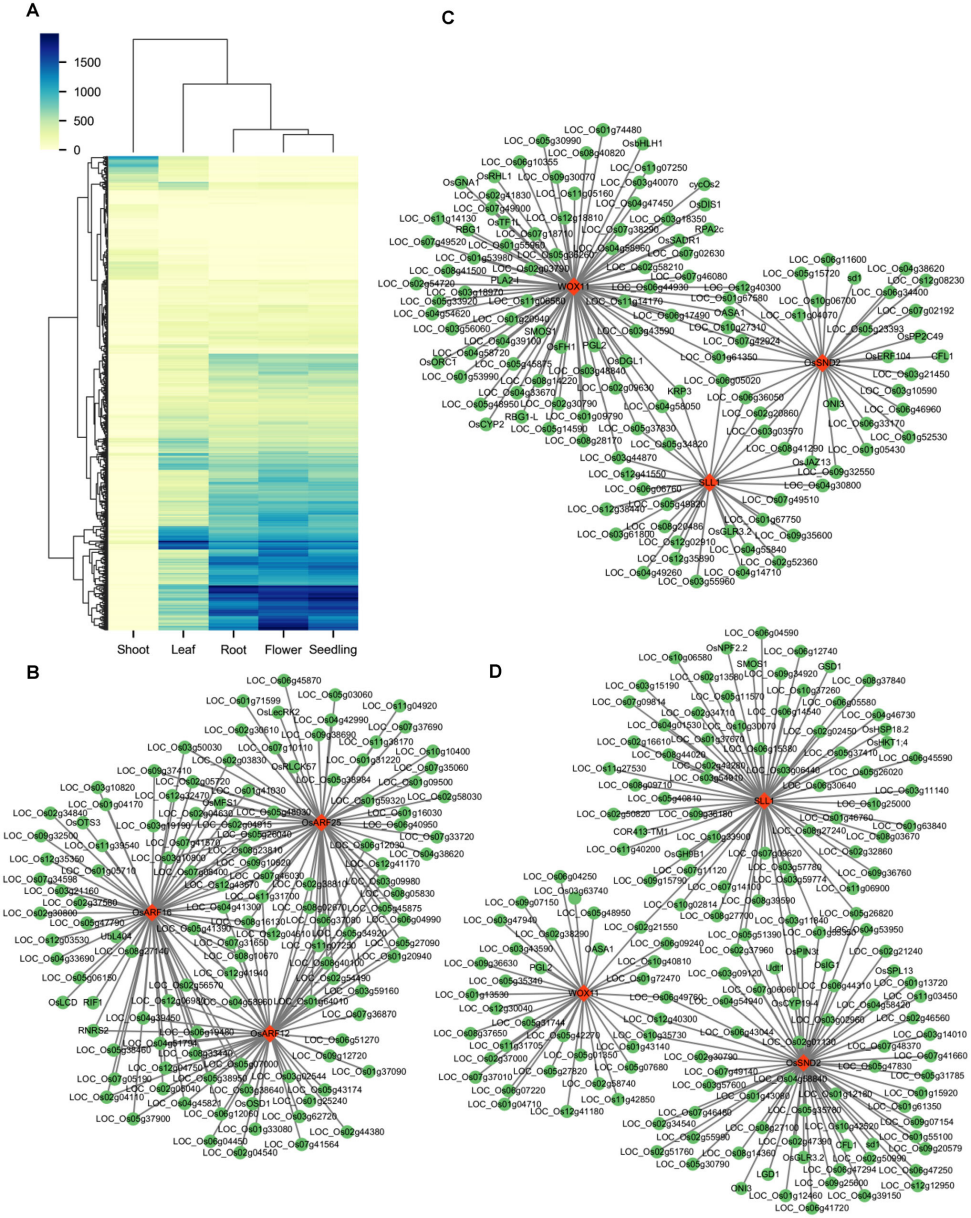
Inference of tissue-specific regulons in rice using InferReg. **A** Clustered heatmap of the number of TGs in regulons of five rice tissues. **B** Regulons of three ARF family TFs inferred from rice roots. **C** Regulons of three TFs, WOX11, OsSND2, and SLL1, inferred from rice roots. **D** Regulons of three TFs, WOX11, OsSND2, and SLL1, inferred from rice leaves

We conducted a detailed analysis of tissue-specific regulation and identified 529 regulons. We noticed noteworthy variations in the number of TGs regulated by the same TF across different tissues (Fig. 6A). These results show that InferReg is useful for predicting gene regulatory relationships and identifying tissue-specific regulons in other plant species (Supplementary Table S6A, B).

After identifying the specific sets of genes, we created gene regulatory networks to understand how these genes regulate each other within different tissues. In the root gene sets, we found several highly correlated key regulatory genes: OsARF12, OsARF25, and OsARF16 (Fig. 6B). These genes are all part of the ARF family, which is known for their role in auxin signaling. Previous studies have shown that mutations in OsARF12 and the double mutant OsARF12/OsARF25 exhibit short root phenotypes, indicating their regulatory role in root elongation (Qi et al. 2012). Knockout mutants of OsARF16 display an auxin-insensitive phenotype (Shen et al. 2015). Auxin is closely linked to cell division, differentiation, and the growth of root hairs, affecting their number and length, indicating its regulatory role in rice root development. Figure 6B illustrates that OsARF12, OsARF25, and OsARF16 have many common target genes (TGs), such as LOC_Os04g41300, LOC_Os08g16130, and LOC_Os11g31700. In addition, we identified regulons that have many TGs in the root but fewer in other tissues. We also created a regulatory network using WOX11 as an example (Fig. 6C). In the root, WOX11 interacts with critical genes such as OsGNA1, OsFH1, and OsDGL1, which are crucial for root elongation (Huang et al. 2013; Jiang et al. 2005; Qin et al. 2013). Furthermore, WOX11 interacts with genes that are involved in the formation of root hair and the development of lateral root, such as OsRHL1 and OsCYP2 (Kang et al. 2013; Kim et al. 2017). In other tissues, WOX11 only interacts with a small number of genes. All of these are part of the identified regulons, indicating the critical regulatory role of WOX11 in root growth and the ability of InferReg to identify tissue-specific regulatory relationships.

Our investigation also assessed the opposite scenario by identifying regulons with a small number of TGs in the root but a large number in the leaves. OsSND2 is linked with leaf development (Ye et al. 2018). In the leaves, OsSND2 interacts with multiple TFs and TGs related to leaf morphology, such as SLL1 (TF), OsYAB1, ONI3, and CFL1 (Jang et al. 2004; Wu et al. 2011) (Fig. 6D). The genes mentioned are also part of the identified regulons. In summary, even though the ChIP-seq data for TFs in rice is relatively limited compared to *Arabidopsis*, InferReg still produced valuable results in rice, validating the effectiveness and applicability of the pipeline.

## Discussion

We have developed the InferReg pipeline, which is a powerful tool for identifying tissue-specific regulons in *Arabidopsis* and *Oryza.* This pipeline not only provides a way to study plant regulons but also ranks the importance of these regulons in each tissue. It highlights the significant potential of GCN in managing graph-structured data, especially in intricate biological networks. To showcase its versatility, we utilized InferReg in rice, broadening its potential applications, and the outcomes were highly promising. We used 833 rice transcript profiles and available ChIP-seq data to identify biologically significant regulons. These tissue-specific regulons played a key role in constructing a gene regulatory network and offered insights into the tissue-specific functions of hub genes in the network.

Both bulk and single-cell techniques can be used to study biological problems from perspectives of transcriptomics, epigenetics, and proteomics. However, their data differ significantly and are suitable for different application scenarios. Bulk data reveal the average characteristics of the overall cell population, such as gene expression levels and protein content. It has high data throughput and low noise, making it suitable for studying the biological processes of living individuals. Due to the maturity of bulk technology and the accumulation of large amounts of data on animals and plants in public databases, it is also suitable for mining patterns of life activities from public data at the species level. The analysis of single-cell data uncovers differences among individual cells and provides detailed information about each cell. This technology is useful for studying cell subpopulations, their varied functions, as well as dynamic processes like cell development, differentiation, and tumor heterogeneity. This research aims to identify essential genes specific to tissues by analyzing the regulatory relationships across different tissues within species using publicly available data. This will help enhance the efficiency of material and energy utilization in breeding. As a result, bulk data are more suitable for this study.

In theory, combining bulk and single-cell expression profiles can help overcome their limitations and provide more comprehensive and accurate information. However, this integration also presents significant challenges in practice, primarily due to two main aspects. First, the inherent data structures and characteristics of bulk RNA-seq and scRNA-seq data differ, making it difficult to map to the identical multidimensional space for further analysis. In some studies, both single-cell and bulk transcriptional profile data are combined to identify crucial cancer genes (Bao et al. 2024; Chen et al. 2022). Essentially, these studies utilize both bulk RNA-seq and scRNA-seq data to identify cancer candidate genes, but they do not take advantage of the correlations between the two types of data at the feature level. Second, the study of biological issues at the tissue and cellular levels can be approached using bulk RNA-seq and scRNA-seq, each with different motivations. Single-cell data integration has gained attention, particularly the fusion of scRNA-seq and scATAC-seq data, which has made significant progress (Cao and Gao 2022; Stuart et al. 2019; Zhang et al. 2022). Single-cell sequencing is capable of detecting various omics in humans and animals, including transcriptomics, chromatin accessibility, and DNA methylation omics. While single-cell remains predominant in plants, there are only a few applications in other single-cell omics in this context. As these technologies become more widely applied in plants and data continue to accumulate, the integration and analysis of different single-cell omics data is expected to provide a more comprehensive understanding of gene regulatory status within cells and reveal regulatory mechanisms.

The InferReg pipeline for predicting tissue-specific regulon offers promising opportunities, but there are still some difficulties that need to be considered. First, the InferReg pipeline combines RNA-seq, TFBS, and ChIP-seq data. RNA-seq data is used to identify potential regulatory relationships between genes, while TFBS and ChIP-seq data provide further evidence to validate these connections. Obtaining RNA-seq data from multiple tissues is relatively straightforward for model plants like *Arabidopsis*, rice, and maize, but it can be more difficult for many other plant species. Additionally, using a smaller number of input transcriptional profiles may reduce the performance of InferReg, although it will not cause a drastic decline. Therefore, it is recommended to use as much RNA-seq data as possible, including various tissues. Additionally, evaluation methods for InferReg performance are limited outside of *Arabidopsis* due to the lack of ground-truth regulatory relationships. ChIP-seq is a widely accepted experimental technique for regulation between transcription factors and target genes. However, the initial step involves designing an antibody that can highly specifically bind to target proteins without non-specific binding or cross-reactivity. As there is limited direct evidence validated through ChIP-seq, we had to rely on indirect evidence from existing literature to evaluate InferReg.

Our study introduces a new tool for studying tissue-specific regulons in plants. This is essential for understanding different biological processes in plants, such as tissue differentiation, development, and stress responses. Furthermore, it helps us understand how certain transcription factors control gene expression in specific organs and tissues, leading to improvements in plant characteristics.

## Materials and methods

### Data collection and processing

In this study, the utilized reference genome sequence was the TAIR10, which represents the genome of the *Arabidopsis* ecotype Columbia (Col-0) (Lamesch et al. 2012). The transcriptome data was obtained from the Plant Public RNA-seq database (Yu et al. 2022). Datasets with a minimum read count of 1,000,000 and a unique alignment rate of at least 0.8 were selected (Conesa et al. 2016). After filtering steps (Supplementary Note S1), 3400 datasets remained, each containing the expression of 37,886 genes. The final dataset consisted of 304, 837, 886, 917, and 456 samples for flower, leaf, root, seedling, and seed, respectively. We obtained 1717 TFs distributed across 58 families for *Arabidopsis* from PlantTFDB (Jin et al. 2016), along with ChIP-seq data for 256 *Arabidopsis* TFs from the ReMap2022 database (Hammal et al. 2022). TFBS information was collected from the PlantTFDB, Cis-BP (Weirauch et al. 2014), and Jaspar databases (Castro-Mondragon et al. 2022).

### Determining TF binding preferences through TFBS analysis

We defined the upstream region of the transcription start site (TSS), extending 2000 bp, as the potential binding region for TFs. To analyze TF binding preferences, we used two commonly used motif scanning tools: FIMO (Grant et al. 2011) and Cluster Buster (Frith et al. 2008). We scanned for motifs within this region and identified potential TF-TG regulatory relationships. We used the default parameters for FIMO and set the parameters for Cluster Buster to -f 5 -c 5 -m 6. Regulatory relationships confirmed by both tools were retained for further analysis.

### Graph neural network

The graph convolutional network captures the high-order neighborhood information of gene regulatory relationships (Zhuang and Ma 2018). The framework is derived from the preliminary regulatory network filtered using multiple omics data. It consists of three main parts:

1. Feature normalization and dimensionality reduction: due to the high dimensionality and sparsity of the gene expression features, we performed feature normalization and dimensionality reduction to standardize the input features of varying lengths. Specifically, we employ adaptive one-dimensional convolution to unify the size of the input features, followed by dimensionality reduction using fully connected layers.
2. Node embedding generation: two-layer GCNs generated node embedding representations based on the graph structure data and the dimensionality-reduced node features. The outer product of the embedding features of the source and target nodes represents the edge embedding.
3. Feature extraction: a convolution neural network (CNN) extracts features from each edge embedding (Supplementary Fig. 1).

The issue has now become a binary link prediction problem. This involves predicting whether an edge $\left(t,u\right)$ is present or absent in the network, where $t$ represents the TF node and $u$ represents the TG node. To balance the number of positive and negative samples, we used a random sampling approach to select negative samples from non-existent edges. The number of negative samples was equal to the number of positive samples. Specifically, assuming $G=\left(V,E\right)$ represents the graph structure of a network, where $V=\left\{{v}_{1},{v}_{2},\dots ,{v}_{N}\right\}$ is the set of nodes, and $E\subseteq V\times V$ is the set of edges. For each TF $t\in V$, let $N\left(t\right)=\left\{v\in V:\left(t,v\right)\in E\right\}$ be the set of neighboring nodes of $t$, and $\left|N\left(t\right)\right|$ represents the degree of $t$. Let $L$ be the set of all edges in $G$. ${L}_{P}$ represents the positive class edges, i.e., ${L}_{P}=\left\{\left(t,u\right)\in L:t\text{ regulates }u\right\}$, and ${L}_{N}=L\backslash {L}_{P}$ represents the non-existent edges. For each TF $t\in V$, randomly select a subset $S\left(t\right)\subseteq V-N\left(t\right)-\left\{t\right\}$ of the same size as $\left|N\left(t\right)\right|$, and replace each $\left(t,u\right)\in {L}_{N}$ with $\left(t,v\right)$, where $v\in S\left(t\right)$. We now have an equal number of negative and positive class edges. The input for the two-dimensional CNN consists of the embedding representation $e\left(t,u\right)$ of the edges, and the labels are assigned as either positive or negative edges. To train the model, we randomly divide all positive and negative samples into training, testing, and validation sets with a ratio of 8:1:1.

Our goal is to minimize the cross-entropy loss function as part of the optimization process. We use the Adam optimizer with a learning rate 0.005 and a weight decay 0.0001 to update the parameters. To prevent overfitting, we included a dropout layer after each feature propagation layer and applied L2 regularization. We also use an early stopping strategy, where the training process halts after 200 consecutive iterations. Additionally, we assess the performance using various metrics such as accuracy, precision, recall, F1 score, and AUC. We evaluate different hyperparameter combinations through cross-validation and select the optimal combination as the parameter setting for the final GCN.

### Threshold-based post-processing

We implemented a threshold-based post-processing approach to enhance the accuracy of our predicted results by reducing false positive regulatory relationships. By default, we used a classification threshold of 0.5 to differentiate between positive and negative samples. In order to uncover more precise regulatory relationships, we adjusted the threshold from 0.5 to 1.0 in increments of 0.1. We noticed that precision improved gradually, while the other three metrics showed a trend of initially showing a slight decline followed by a sharper drop. As a result, we identified the threshold at which the F1 score decreased to 0.8 as the optimal threshold (threshold = 0.77).

### Differential expression analysis

For the five tissue types, we conducted a differential expression analysis and focused on identifying upregulated genes. To do this, we used the Voom method in R package limma (Ritchie et al. 2015). First, we created an expression matrix for a linear model, with tissue type as the explanatory variable. Then, we applied the Voom method to transform the gene expression matrix into log counts per million (log CPM) data, which is suitable for linear modeling. Next, we constructed a contrast matrix to define pairwise comparisons among the five tissue types. Subsequently, we used functions lmFit and contrasts fit in the limma package to fit the model and perform differential expression testing based on the contrast matrix. We then utilized the eBayes function was employed to estimate the parameters, calculate statistical measures, and determine the corresponding *p*-value for differential expression. The DEGs were extracted using the false discovery rate (FDR) less than 0.01 and the log-fold change (log |FC|) greater than 1 as the criteria.

### Construction of GO gold standard data

To create the Gene Ontology (GO) gold standard, we followed these steps (Ferebee and Buckler 2023). First, we gathered all the GO terms for *Arabidopsis* from the GO database. Then, we singled out the GO terms that were associated with specific tissues. These terms describe various biological processes associated with tissue development, morphogenesis, and environmental responses. Subsequently, we collected the genes annotated with these tissue-related GO terms. This gene set represents the central functional genes of the specific tissues. We have designated this set of genes as the GO gold standard, which will be used as a benchmark to assess the accuracy of our pipeline. When choosing GO annotations, we only considered entries with evidence codes such as EXP (expression evidence), IMP (inferred from mutant phenotype), IDA (inferred from direct assay), IPI (inferred from physical interaction), IGI (inferred from genetic interaction), IEP (inferred from expression pattern), TAS (traceable author statement), NAS (non-traceable author statement), and IC (inferred by curator) to ensure reliability of annotations.

For the purpose of identifying functionally relevant gene sets highly associated with different tissues, we conducted searches using tissue-specific keywords related to GO annotations to retrieve relevant genes. Table 3 displays GO keywords searched for in other tissues.

**Table 3:** TFs associated with a specific tissue extracted by querying GO annotation keywords related to that tissue

Tissue	GO keywords	TF numbers
Flower	Flower, floral, inflorescence, anther, stamen, pistil, carpel, petal, sepal, pollen, pollination, fertilization	94
Leaf	Leaf, foliage, photosynthesis, chloroplast, stomata, mesophyll, cuticle	89
Root	Root, xylem, phloem, vascular, trichoblast, trichome, vasculature, stele, tracheary, procambium, sieve	93
Seed	Seed, embryo, endosperm, aleurone, maturation, testa, embryogenesis	127
Seedling	Seedling, cotyledon, hypocotyl, epicotyl, germination, plumule, radicle, shoot	139

### Prioritization of regulons

We used five metrics to prioritize regulons, including out-degree, betweenness centrality, closeness centrality, and GO functional enrichment of the regulons (Ferrari et al. 2022). The first three metrics were calculated using the Networkx library (Hagberg et al. 2008) and were used to evaluate the importance of each TF in regulating its targets.

The Borda count method was used to combine the rankings from network topology and GO functional enrichment (Supplementary Note S1). This method is a multi-criteria decision-making approach where each regulon is given a rank for each metric, and these ranks are combined to give a Borda score for each regulon. This approach helps in a thorough assessment of multiple metrics and comprehensive prioritization of the regulons.

### Acquisition of phenotype-related genes in five *Arabidopsis* tissues

We used the AraGWAS database to find genes linked to the phenotypes of five specific tissues in *Arabidopsis*: leaf, flower, root, seed, and seedling. We set two filters: a Bonferroni-corrected *p*-value less than 0.001 and a Minor Allele Count greater than 5. These strict filters were used to reduce the chance of random findings and misinterpretation.

From the chosen studies, we focused on single nucleotide polymorphisms (SNPs) located within the coding regions. We chose this because SNPs in coding regions directly affect the protein sequence and may influence the phenotypes. We speculated that genes within these regions would strongly relate to their respective tissue-specific phenotypes.

## Supplementary Information

Below is the link to the electronic supplementary material.

Supplementary file1 (XLSX 154 KB)

Supplementary file2 (XLSX 21 KB)

Supplementary file3 (XLSX 3649 KB)

Supplementary file4 (XLSX 12 KB)

Supplementary file5 (XLSX 56 KB)

Supplementary file6 (XLSX 13294 KB)

Supplementary file7 (XLSX 9018 KB)

Supplementary file8 (XLSX 4281 KB)

Supplementary file9 (JPG 682 KB)

Supplementary file10 (DOCX 17 KB)

Supplementary file11 (DOCX 22 KB)

## Data Availability

The code is freely available at https://github.com/shengtudai2/InferReg. The datasets can be downloaded at 10.5281/zenodo.11176206.
