# Correction: Inference and prioritization of tissue-specific regulons in *Arabidopsis* and *Oryza*

**DOI:** 10.1007/s42994-024-00191-3

**Published:** 2024-12-19

**Authors:** Honggang Dai, Yaxin Fan, Yichao Mei, Ling-Ling Chen, Junxiang Gao

**Affiliations:** 1 Hubei Key Laboratory of Agricultural Bioinformatics, College of Informatics, Huazhong Agricultural University, Wuhan, 430070 China; 2 State Key Laboratory for Conservation and Utilization of Subtropical Agro-Bioresources, College of Life Science and Technology, Guangxi University, Nanning, 530004 China

**Correction: aBIOTECH (2024) 5:309-324** 10.1007/s42994-024-00176-2

The article Inference and prioritization of tissue-specific regulons in Arabidopsis and Oryza, written by Honggang Dai, Yaxin Fan, Yichao Mei, Ling-Ling Chen, Junxiang Gao, was originally published Online First without Open Access. After publication in volume 5, issue 3, pages 309–324 the authors decided to opt for Open Choice and to make the article an Open Access publication. Therefore, the copyright of the article has been changed to © The Authors 2024 and the article is forthwith distributed under the terms of the Creative Commons Attribution CC BY 4.0 International License, which permits use, sharing, adaptation, distribution and reproduction in any medium or format, as long as you give appropriate credit to the original author(s) and the source, provide a link to the Creative Commons licence, and indicate if changes were made.

The images or other third party material in this article are included in the article’s Creative Commons licence, unless indicated otherwise in a credit line to the material. If material is not included in the article’s Creative Commons licence and your intended use is not permitted by statutory regulation or exceeds the permitted use, you will need to obtain permission directly from the copyright holder.

To view a copy of this licence, visit http://creativecommons.org/licenses/by/4.0/.

