# Supplementary figures and images for: Inference and prioritization of tissue-specific regulons in Arabidopsis and Oryza
Source: aBIOTECH. 2024 Jul 16;5(3):309–24. doi: 10.1007/s42994-024-00176-2 (PMC11399499; doi:10.1007/s42994-024-00176-2)

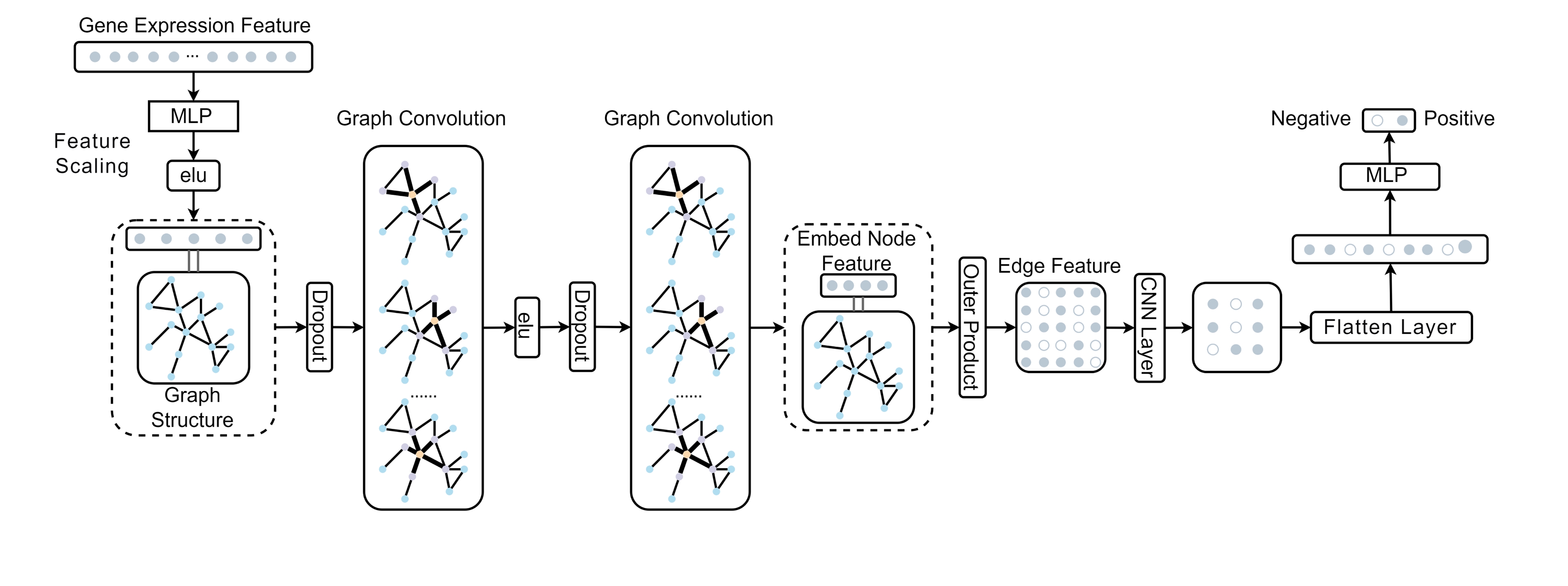

Supplement: Supplementary file 9 — Supplementary file9 (JPG 682 KB) [file 42994_2024_176_MOESM9_ESM.jpg]
