# Supplementary material for: Inference and prioritization of tissue-specific regulons in Arabidopsis and Oryza
Source: aBIOTECH. 2024 Jul 16;5(3):309–24. doi: 10.1007/s42994-024-00176-2 (PMC11399499; doi:10.1007/s42994-024-00176-2)
Supplement: Supplementary file 10 — Supplementary file10 (DOCX 17 KB) [file 42994_2024_176_MOESM10_ESM.docx]

**Supplementary figure legend**

**Supplementary Fig. S1.** The Graph Neural Network (GNN) architecture in the InferReg framework. This diagram explains how the model processes the raw regulatory relationships and gene expression matrices. The gene expression data undergoes initial processing using a Multilayer Perceptron (MLP) with the Exponential Linear Unit (elu) activation function to standardize node feature dimensions. The GNN then applies two Graph Convolutional Networks (GCN) layers to the graph-structured data, enhanced with node features, to extract embedded representations for each node. Edge embeddings are computed using the outer product of node embeddings. These embeddings are further refined through a two-dimensional Convolutional Neural Network (2D CNN) and a flattened layer. Finally, the model classifies the edges as positive or negative using another MLP.
