# Supplementary material for: Inference and prioritization of tissue-specific regulons in Arabidopsis and Oryza
Source: aBIOTECH. 2024 Jul 16;5(3):309–24. doi: 10.1007/s42994-024-00176-2 (PMC11399499; doi:10.1007/s42994-024-00176-2)
Supplement: Supplementary file 11 — Supplementary file11 (DOCX 22 KB) [file 42994_2024_176_MOESM11_ESM.docx]

**Supplementary Note S1: Details of the Method**

## Processing of expression data

The gene expression matrix comprises $m$ genes and $n$ samples, with values denoted as $x_{ij}$, where $1\leq i\leq m$ and $1\leq j\leq n$. To process high-expression outliers in the matrix, we take the following steps.

(1) Calculate the mean $\mu$ and standard deviation $\sigma$ of $x_{ij}$ (Equations 1 and 2):

| $\begin{aligned} \mu=\frac{1}{mn}\sum_{i=1}^{m} \sum_{j=1}^{n} x_{ij} \end{aligned}$ | (1) |
| --- | --- |
| $\begin{aligned} \begin{aligned} \sigma=\sqrt{\frac{1}{mn}\sum_{i=1}^{m} \sum_{j=1}^{n} \left( x_{ij}-\mu\right)^{2}} \end{aligned} \end{aligned}$ | (2) |

(2) Set a threshold $t$ for outliers (Equation 3):

| $\begin{aligned} t=\mu+3\sigma\end{aligned}$ | (3) |
| --- | --- |

(3) Any gene expression value exceeding this threshold was marked as an outlier, and the outliers were transformed using Equation 4.

| $\begin{aligned} \begin{aligned} f'_{i}=t+ln\left( f_{i}-t+1 \right) \end{aligned} \end{aligned}$ | (4) |
| --- | --- |

where $f_{i}$ represents the original gene expression value and $f'_{i}$ represents the transformed gene expression value. This transformation helps to avoid biases introduced by outliers and ensures that all expression values are positive.

Subsequently, we used the following three criteria to filter out low expression genes to improve the accuracy of downstream analyses. (i) Genes with an average FPKM value below 2 across all samples were excluded. (ii) Genes with zero expression values in more than half of the samples were also excluded. (iii) A coefficient of variation (CV) filter was applied to genes with relatively stable expression across all samples. The CV, which measures variation relative to the mean, was calculated for each gene $i$ using the formula $CV_{i}={\sigma_{i}/\mu}_{i}$, where $\sigma_{i}$ and $\mu_{i}$ represent the standard deviation and mean expression value of gene $i$ across all samples, respectively. Genes with a CV lower than 0.5 were considered low variation and removed from the expression matrix.

## Prioritization of regulons

We analyzed each TG of the regulons for the GO functional enrichment analysis to identify enriched GO terms. The enrichment analysis employed statistical tests, and we selected significantly enriched GO terms with an FDR less than 0.05. We proposed a novel method to assess the importance of regulons in GO enrichment analysis. Initially, we calculated a baseline score for each combination of regulons and GO terms using the following formula:

| $\begin{aligned} \begin{aligned} \begin{aligned} score=\frac{\left( 1-q_{val} \right)\times e_{fold}\times{10}^{-p_{val}}}{f_{size}\times n_{hits}} \end{aligned} \end{aligned} \end{aligned}$ | (5) |
| --- | --- |

In this formula, $q_{val}$ represents the adjusted p-value, which indicates the association between the regulon and the GO term. $e_{fold}$ represents the enrichment fold, which is the ratio of the observed number of genes to the expected number of genes. $p_{val}$ represents the raw p-value, indicating the significance of the difference between the observed number of genes and the expected number of genes. $f_{size}$ is the number of genes regulated by the regulatory element, and $n_{hits}$ is the number of genes in the regulons included in the specific GO term.

Next, a score adjustment was performed for regulons associated with expected GO term enrichments. Specifically, if a regulon was associated with an expected GO term enrichment, the score was multiplied by 10. Finally, each regulon’s total score was calculated by summing the scores for all GO terms. This total score represented the overall importance of the regulons in all GO term enrichments. This approach considered the regulatory ability of regulons, their association with GO terms, the degree of enrichment, and their relevance to expected GO term enrichments, resulting in a comprehensive assessment of the importance of the regulatory elements in GO term enrichment analysis.

## Training and predicting steps

We outline the process for predicting regulatory relationships in *Arabidopsis*, based on Figure 1A of the manuscript.

(1) Create an expression matrix from RNA-seq data and identify potential regulatory relationships using the GRNBoost2 algorithm.

(2) Filter regulatory relationships with TFBS data support from the potential regulations mentioned above.

(3) Additionally, filter out the regulatory connection between TF and genes confirmed by ChIP-seq data. The result of each of the first three stages is associations between genes, with the distinction being that the dependability gradually improves. The associations between the genes produced in step (3) are backed by ChIP-seq data, and we consider them trustworthy.

(4) We used the regulatory relationships identified in step (3) as positive samples and randomly selected non-existing regulatory relationships as negative samples. We then used these samples to create the training set. After training the GCN model with this dataset, we obtained the parameters of the GCN model. With these parameters, the model can predict the relationships between TFs (transcription factors) and target genes.

(5) During the prediction stage, the regulatory connections between transcription factors (TFs) and target genes from step (3) are fed into the graph convolutional network (GCN) model that was trained in step (4). The GCN then undergoes convolution operations on each gene node to produce an embedding representation of the node. This convolution process involves acquiring the node's own features and aggregating feature data from neighboring nodes.

(6) After obtaining the embedding representation of gene nodes, the embedding representation of the link between two nodes is calculated by taking the outer product of their feature vectors.

(7) Use CNN for binary classification prediction on the links from step (6) to determine their presence.

Our study showcases the effectiveness of the InferReg model in species without ChIP-seq data, using rice as an example. While this can be done through the computational pipeline, its performance should be evaluated on a case-by-case basis. We first collected RNA-seq data from 833 rice samples for step (1), and then utilized 1580 TFBSs from rice for step (2), which produced regulatory relationships supported by TFBS data in rice. As there is a lack of ChIP-seq data for transcription factors in rice, meaning that there's no definitive regulatory relationships, we are unable to create a training set. Therefore, we have to skip steps (3) and (4). Instead, we will input the regulatory relationships identified in step (2) directly into the GCN model trained with Arabidopsis data in step (5). This involves using convolution operations to compute the embedding representation of gene nodes. Subsequently, we will proceed to step (6) to calculate the embedding representation of the connections between each pair of nodes and then move on to step (7) to predict the existence of regulatory relationships.
